# Identification of an HLA-A^*^0201-restricted T-cell epitope derived from the prostate cancer-associated protein prostein

**DOI:** 10.1038/sj.bjc.6601642

**Published:** 2004-03-02

**Authors:** A Kiessling, S Stevanovic, S Füssel, B Weigle, M A Rieger, A Temme, E P Rieber, M Schmitz

**Affiliations:** 1 Institute of Immunology, Medical Faculty, Technical University of Dresden, Fetscherstr. 74, Dresden 01307, Germany; 2 Department of Immunology, Institute for Cell Biology, University of Tübingen, Auf der Morgenstelle 15, Tübingen 72076, Germany; 3 Department of Urology, Medical Faculty, Technical University of Dresden, Fetscherstr. 74, Dresden 01307, Germany

**Keywords:** prostein, T cells, tumour antigen, dendritic cells, immunotherapy

## Abstract

The development of T-cell-based immunotherapies of cancer largely depends on the availability of tumour-associated antigens capable of eliciting tumour-directed cytotoxic T-cell responses. In prostate cancer, the number of antigens defined as suitable targets of cytotoxic T lymphocytes (CTLs) is still limited. Recently, prostein was identified as a transmembrane protein that is highly restricted to prostate tissues. In our study, prostein transcripts were found to be abundant in both malignant and nonmalignant prostate tissue samples. To identify immunogenic CD8+ T-cell epitopes, human leucocyte antigen-A^*^0201-binding peptides were selected from the amino-acid sequence of prostein and were used for the *in vitro* stimulation of CD8+ T lymphocytes. Specific CTLs were raised against the prostein-derived peptide CLAAGITYV that were capable of lysing prostate cancer cells, indicating that this peptide is naturally generated by tumour cells. Our data suggest that prostein is a suitable candidate to be included in a T-cell-based immunotherapy of prostate cancer.

Prostate carcinoma (PCa) is the most common cancer diagnosis and the second leading cause of cancer-related deaths in men (Howe *et al*, 2001). The absence of effective curative therapies for advanced and recurrent prostate tumours has entailed an intensive search for novel treatment modalities.

T cells provide a powerful compartment of adaptive immunity with the potential to survey and respond to a great diversity of antigens. The immunotherapy of human tumours has been put forward by the finding that CD8+ cytotoxic T lymphocytes (CTLs) are capable of effective recognition and destruction of tumour cells (Rosenberg, 1997). Consequently, much attention has been paid to the identification and characterisation of tumour-associated antigens (TAAs) that may provide target structures for a T-cell-based vaccination strategy (Saffran *et al*, 1999; Nelson, 2002).

While searching for new potential immunotherapeutic targets of PCa, a number of prostate- or PCa-associated transcripts and proteins have been identified during the last years such as six-transmembrane epithelial antigen of the prostate (Hubert *et al*, 1999), prostase (Nelson *et al*, 1999), prostate androgen-regulated transcript 1 (PART-1) (Lin *et al*, 2000), human novel prostate-specific antigen (PSA) (Naz *et al*, 2002), human prostate-specific gene-1 (Herness and Naz, 2003) and NKX3.1 (He *et al*, 1997). However, recent observations suggest that the expression of some of these molecules is not strictly confined to prostate tissue (Hubert *et al*, 1999; Yousef *et al*, 1999; Sidiropoulus *et al*, 2001). The suitability of these molecules for T-cell-based immunotherapy is limited, since targeting epitopes that are presented on essential tissues may induce adverse autoimmune reactions. Other molecules are downregulated in tumour tissue when compared with normal prostate tissue (Bowen *et al*, 2000; Xu *et al*, 2003).

The high diversity, the genetic instability and the escape mechanisms of tumours determine the number of individual target structures for T-cell-based immunotherapeutic strategies. In addition, the number of prostate-specific proteins known to elicit T-cell responses is still limited. Therefore, the identification of new TAAs can facilitate T-cell-based vaccination strategies in PCa patients. The efficiency of the antitumour response could be increased by including a collection of well-characterised epitopes derived from diverse antigens for the activation of patients' T cells.

Recently, prostein was identified as a novel prostate-specific protein using a cDNA library substraction strategy (Xu *et al*, 2001). The expression of prostein is highly restricted to malignant and nonmalignant prostate tissues at both mRNA and protein level.

Having confirmed the expression of prostein in normal and malignant prostate tissues by real-time PCR, we investigated the suitability of prostein to serve as a target antigen for PCa-directed CTLs. An immunogenic human leucocyte antigen (HLA)-A^*^0201-restricted peptide derived from prostein was identified, which proved to be effective in activating tumour-directed CTL responses. The newly identified peptide may be appropriate to be included as a target structure in a T-cell-based immunotherapy of patients with HLA-A^*^0201-positive PCa.

## MATERIAL AND METHODS

### Cell lines

The PCa cell lines LNCaP 1740 and PC-3, the mutant cell line T2 and the chronic myelogenous leukemia cell line K562 (all from American Type Culture Collection, Manassas, VA, USA) were cultured according to the manufacturer's instructions. The melanoma cell line 93.04A12.1 was kindly provided by Dr CJM Melief (University Hospital, Leiden, The Netherlands). This cell line was maintained in RPMI 1640 medium (Biochrom, Berlin, Germany) supplemented with 2 mM L-glutamine, 1% nonessential amino acids (both from Biochrom), 100 *μ*g ml^−1^ penicillin, 100 *μ*g ml^−1^ streptomycin (both from Life Technologies, Karlsruhe, Germany) and 10% heat inactivated foetal calf serum (FCS) (Biochrom).

The androgen deprivation and stimulation experiments were performed as described previously (Xu *et al*, 2001). Briefly, LNCaP 1740 cells were cultured for 24 h in phenol red-free RPMI (Life Technologies) supplemented with 1% nonessential amino acids, 10 mM HEPES (Life Technologies) and 10% charcoal-stripped FCS (Biochrom). The cultivation was continued for additional 48 h in the androgen-depleted medium or in the presence of 1 nM of the synthetic androgen R1881 (Perkin-Elmer Life Sciences, Rodgau-Jügesheim, Germany). Subsequently, the cells were harvested and were used as target cells in a chromium release assay.

### Prostate cancer patients and tissue samples

All tissue and blood samples were obtained from prostatectomised PCa patients and healthy donors with informed consent. All samples from PCa patients were obtained together with clinical data and pathological classification (Table 1

**Table 1:** Pathological and clinical parameters (UICC TNM classification system from 1997) and the percentage of tumour cells in tissue samples analysed by real-time PCR

**Patient**	**Age (years)^a^**	**TNM classification**	**G**	**GS**	**Percentage of tumour cells (%)^b^** **malignant/nonmalignant sample**
1	62	pT2 pN0 cM0	IIb	7	60/0
2	62	pT2a pN0 cM0	NA	NA	70/0
3	63	pT2b pN0 cM0	IIb	6	85/0
4	61	pT2b pN0 cM0	IIIb	8	100/0
5	62	pT2b pN0 cM0	IIa	7	80/0
6	63	pT2b pN0 cM0	IIIa	7	90/0
7	59	pT2b pN0 cM0	IIb	7	85/10
8	60	pT2b pN0 cM0	IIb	6	80/0
9	66	pT3 pN0 cM0	NA	8	95/0
10	71	pT3b pN1 cM0	IIIa	7	70/0
11	59	pT3a pN0 cM0	IIIb	9	95/0
12	73	pT3a pN0 cM0	IIIa	8	80/0
13	58	pT4 pN0 cM0	IIb	7	75/0
14	61	pT4 pN0 cMx	IIb	6	75/0
15	57	pT4 pN1 cM0	IIIb	9	75/0

a At the time point of prostatectomy.

b Percentage of tumour cells within the epithelial cells. G, tumour grade; GS, Gleason score; N, lymph node metastases; NA, not available; p, pathological examination; T, tumour stage.

). We analysed pairs of tissue samples (specimens of primary PCa and autologous nonmalignant prostate tissue) from 15 patients.

### RNA isolation and cDNA synthesis

Total RNA was extracted by standard procedures (Trizol LS Reagent; Life Technologies, Karlsruhe, Germany) and quality was analysed by agarose-gel electrophoresis. After DNA digestion (DNase I; Amersham Pharmacia Biotech, Freiburg, Germany), cDNA synthesis was performed using 4 *μ*g of total RNA and random hexamer primers (Ready to Go You Prime First Strand Kit; Amersham Pharmacia Freiburg Biotech, Germany).

### Quantitative reverse transcription (RT)–PCR

The mRNA quantity of prostein was determined using a LightCycler (LC)-based real-time PCR protocol based on SYBR Green I detection (LC – FastStart DNA Master SYBR Green I; Roche Diagnostics, Mannheim, Germany) and the primer pair Prostein_N1 (5′-AACCTTTGGCCTGGAGGTGTGTTTG-3′) and Prostein_C1 (5′-GGGATGAGAAAGAGGCTCAGCAGGA-3′) to amplify a 240 bp cDNA fragment spanning two exons.

The mRNA copy number was adjusted to the RT–PCR product quantity of hypoxanthine phosphoribosyltransferase (HPRT) determined by LC technique using the primer pair HPRT_N1 (5′-CCCTGGCGTCGTGATTAGTGATGAT-3′) and HPRT_C1 (5′-TGCTTTGATGTAATCCAGCAGGTCAGC-3′) for the amplification of a 238 bp fragment.

Serial dilutions of plasmid DNA containing the prostein or HPRT fragments over six log scales (10^1^–10^6^ molecules capillary^−1^), allowing the linear regression of the crossing points *vs* the logarithm of sample concentration (regression coefficient: −1.00; mean squared error: <0.15), were used as internal template standards for the calculation of the transcript copy number (calculation via fit point mode of the LC quantification software version 3.5; Roche Diagnostics, Mannheim, Germany). The detection limit of the assays was 10 transcript copies. Each determination was carried out twice for each cDNA sample. From the mean values, the molecule ratios of prostein to HPRT transcripts were calculated.

### Epitope prediction and peptide synthesis

Potential HLA-A^*^0201 ligands were selected from the amino-acid sequence of prostein (accession no. NP_149093) using a matrix pattern suitable for the calculation of peptides fitting to an HLA-A^*^0201 motif (Rammensee *et al*, 1999; http://www.syfpeithi.de). This scoring system allows the prediction of the binding affinity of a given peptide to HLA-A^*^0201 by evaluating each amino acid within the sequence for their preference in the respective position. The allocation of values is based on the frequency of the respective amino acid in known natural ligands, T-cell epitopes or binding peptides. The six highest scoring peptides were synthesised as described previously (Kiessling *et al*, 2002).

### Competition assay

Binding studies of potential HLA-A^*^0201 fitting peptides were carried out using the B-cell lines LCL721 or JY and a fluorescence-based competition assay, essentially as described by van der Burg *et al* (1995), but without performing acid strip. Reporter peptide was ILK(FITC)EPVHGV from HIV-1 reverse transcriptase and positive control was YLLPAIVHI from RNA helicase p72. Fluorescence intensities were recorded by flow cytometry (FACSCALIBUR; Becton Dickinson, Heidelberg, Germany).

### *In vitro* generation of prostein-specific CD8+ cytotoxic T lymphocytes

Briefly, peripheral blood mononuclear cells were prepared from blood samples by Ficoll–Hypaque (Biochrom) density centrifugation. Monocytes were isolated by immunomagnetic cell separation with an anti-CD14 antibody coupled to paramagnetic microbeads (Miltenyi Biotech, Bergisch Gladbach, Germany) according to the manufacturer's instructions. Mature monocyte-derived dendritic cells (DCs) were generated as described previously (Schmitz *et al*, 2000).

To generate prostein-specific CTLs, mature DCs were either pulsed with a cocktail of the prostein-derived peptides 1478, 1479 and 2004 or a cocktail of the peptides 1472, 1487 and 1494 at a concentration of 20 *μ*g ml^−1^ of each peptide in serum-free RPMI 1640 medium for 3 h. After washing 2 × 10^5^ peptide-loaded DCs were cocultured with 2 × 10^6^ immunomagnetically isolated CD8+ T cells well^−1^ of a 24-well tissue culture plate (Greiner, Frickenhausen, Germany). T-cell cultures stimulated with DCs pulsed with the peptides 1478, 1479 and 2004 were established from two prostate cancer patients (donors 1 and 3) and a healthy individual (donor 2). The peptides 1472, 1487 and 1494 were used to stimulate an additional culture of donor 2 and T cells obtained from another patient (donor 4). The T cells were cultured in 2 ml RPMI 1640 medium well^−1^ supplemented with 10% human serum (CCpro, Neustadt, Germany), 100 U ml^−1^ IL-2 and 10 ng ml^−1^ IL-7 (both from Strathmann Biotech, Hanover, Germany). After 7 days, cultures were washed and restimulated with peptide-loaded DCs at a responder to stimulator ratio of 5 : 1. After three rounds of weekly restimulation, the cultures were tested for the presence of prostein-specific CTLs.

### Chromium release assay

Cytotoxic activity of the *in vitro*-stimulated CTLs was assayed against the HLA-A^*^0201-positive mutant cell line T2 pulsed with the individual prostein-derived peptides or an irrelevant HLA-A^*^0201-binding peptide from HIV-1 reverse transcriptase at a concentration of 50 *μ*g ml^−1^, against the PCa cell line LNCaP 1740, the melanoma cell line 93.04A12.1 and against K562 cells as targets in a 4 h standard ^51^Cr release assay as described previously (Kiessling *et al*, 2002). The HLA-A2 restriction of CD8+ T-cell-mediated lysis was tested at an effector cell to target cell ratio of 30 : 1 in the presence of the anti-HLA-A2 monoclonal antibody derived from the MA2.1 hybridoma (American Type Culture Collection) at a final concentration of 1 *μ*g ml^−1^.

### Flow cytometry

To evaluate the expression of HLA-A2 on the tumour cell lines used as target cells in the chromium release assays, LNCaP 1740 cells and the melanoma cell line 93.04A12.1 were harvested and resuspended in FACS buffer (phosphate-buffered saline+0.05% sodium acid). Thereafter, the cells were incubated with the anti-HLA-A2 monoclonal antibody MA2.1 or an isotype-matched control antibody (Mouse IgG1; BD Pharmingen, San Diego, CA, USA) both at a concentration of 10 *μ*g ml^−1^ and stained with a PE-labelled goat anti-mouse IgG F(ab′)_2_ fragment (Beckman Coulter, Krefeld, Germany), followed by flow cytometric analysis. To verify the specificity of MA2.1, the HLA-A2-negative PCa cell line PC-3 was included in the FACS analysis (Figure 3C).

## RESULTS

### Quantitative assessment of prostein mRNA expression in prostate tissue specimens

**Figure 1:**
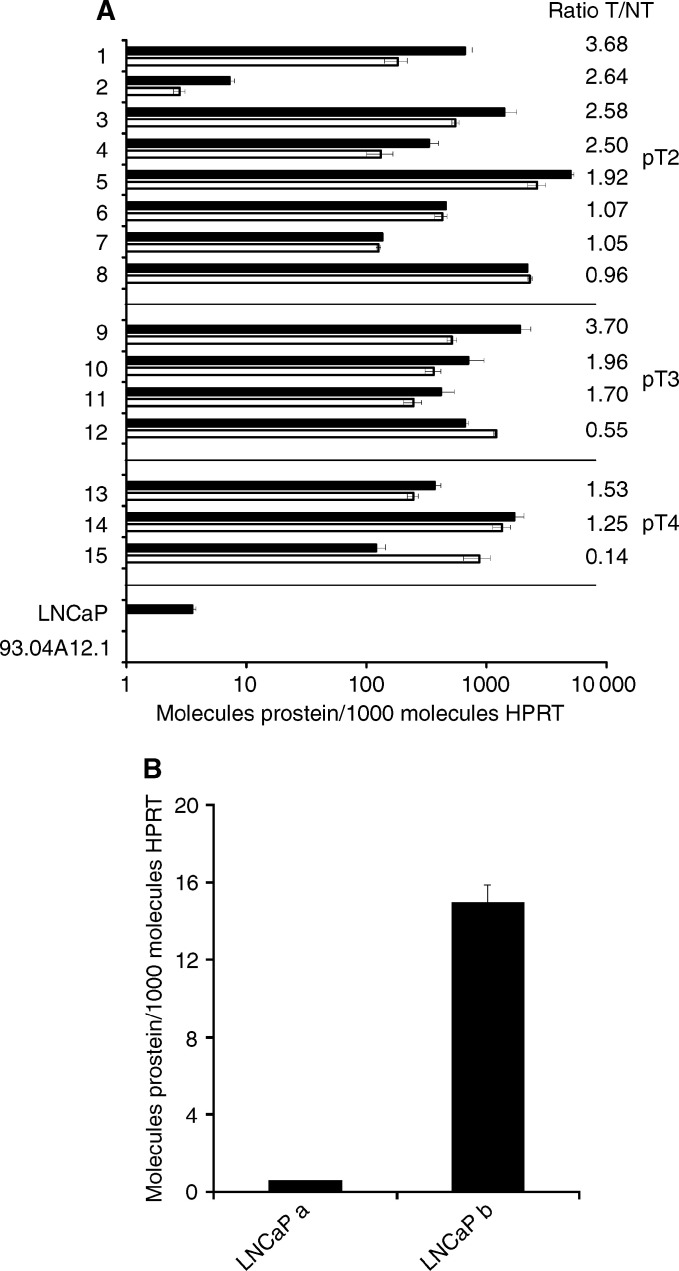
Real-time PCR analysis of prostein mRNA expression in matched samples of malignant and nonmalignant prostate tissues, in the PCa cell line LNCaP 1740, and the melanoma cell line 93.04A12.1. (**A**) To quantify the prostein mRNA expression in 15 paired cDNA samples of tumourous (black bars) and nontumourous (white bars) prostate tissues and in LNCaP 1740 and 93.04A12.1 cells a 240 bp fragment was amplified in a SYBR Green I-based LC assay. The transcript quantity was normalised to the expression level of HPRT. The results represent the means of two LC runs, bars indicate s.e. The ratio of prostein expression in the tumourous related to the transcript quantity in the corresponding nontumourous tissue sample (ratio T/NT) is given for each tissue pair. Patients' data were classified according to their tumour stage (pT) and within the groups according to the T/NT ratios. (**B**) The PCa cell line LNCaP 1740 was cultured for 48 h in the androgen-depleted medium (LNCaP a) or in the presence of 1 nM of the synthetic androgen R1881 (LNCaP b) and then used for RNA preparation and quantification of prostein transcripts. The results represent the means of two LC runs, bars indicate s.e.

Paired malignant and nonmalignant prostate tissue specimens obtained from 15 patients with histologically confirmed PCa were analysed for prostein expression using the quantitative RT–PCR. The pathological and clinical parameters as well as the percentage of tumour cells in the individual samples as determined histologically are summarised in Table 1. As depicted in Figure 1

, prostein mRNA was detected in all tested normal prostate tissue samples, in the corresponding tumours with different degrees of progression and in the PCa cell line LNCaP. The expression level of prostein was highly variable among the patients. In 13 out of 15 patients, prostein transcripts were found at higher or similar levels in the tumour samples when compared with the corresponding nonmalignant tissues (Figure 1A), demonstrating that prostein expression is maintained or even upregulated in the great majority of tumourous tissues. The mean value of all ratios of prostein expression in the tumourous tissue to that in the corresponding nontumourous tissue (T/NT ratio) was 1.82. When the samples were classified according to the pathological stage and grade, we found higher mean T/NT ratios in tumours of early stages and lower grades (Table 2

**Table 2:** Association of tumour stage and grade with the expression level of prostein mRNA as determined by quantitative RT–PCR

	**Tumour stage**			**Gleason score**		**Tumour grade**	
	**pT2 (*n*=8)**	**pT3 (*n*=4)**	**pT4 (*n*=3)**	**⩽7 (*n*=9)**	**⩾7 (*n*=5)**	**IIa, Iib (*n*=7)**	**IIIa, IIIb (*n*=6)**
T/NT ratio^a^	2.05	1.98	0.97	1.78	1.72	1.85	1.32


a Mean value of the individual ratios of transcript quantity in the tumourous samples (T) to that in the corresponding nontumourous tissues (NT) when classified according to pathological stages and grades.

*n*=number of paired tissue samples; RT–PCR=reverse transcription–PCR.

). Owing to the high variability among the T/NT ratios of the individual samples with the same pathological features and a similar percentage of tumour cells, this correlation was not statistically significant (unpaired Student's *t*-test). Furthermore, no correlation between the Gleason score of the tumours and the T/NT ratios was determined (Table 2).

Following the observation by Xu *et al* (2001) that prostein expression is regulated by androgens, the androgen-responsive PCa cell line LNCaP 1740 was grown in medium containing FCS that was depleted of androgens by charcoal stripping or in the same stripped medium supplemented with the synthetic androgen R1881 and then used for the quantification of prostein transcripts. As depicted in Figure 1B, the prostein mRNA expression was downregulated but still detectable under conditions of androgen deprivation when compared with LNCaP cells cultured in the presence of R1881.

### Selection of HLA-A^*^0201-binding peptides

Using a suited algorithm, the amino-acid sequence of prostein was screened for peptides predicted to bind to HLA-A^*^0201, the most common HLA class I allele in Caucasian individuals (Table 3

**Table 3:** Prediction of HLA-A^*^0201-restricted prostein-derived peptides and determination of binding affinity by a competition assay

Peptide	**Position^a^**	**Sequence**	**MW**	**Length^b^**	**Score**	**rBA (%)^c^**
1472	394–402	ILPYTLASL	989.6	9	29	87.5
1478	452–460	GLLPPPPAL	873.5	9	28	55.0
1479	393–402	QILPYTLASL	1117.6	10	30	92.6
1487	255–263	ALLPRLHQL	1059.7	9	31	84.1
1494	507–516	SLFMGSIVQL	1093.6	10	29	89.2
2004	31–39	CLAAGITYV	909.5	9	28	94.0

a The given numbers indicate the position of the peptide in the amino-acid sequence of prostein (GenBank accession no. NP_149093).

b Number of amino acids.

c The relative binding affinities were determined by comparing the inhibition of the reporter peptide binding by the analysed peptides in relation to the inhibition obtained with a positive control peptide which was set as 100%.

The positive control peptide was YLLPAIVHI from RNA helicase p72 and the reporter peptide was ILK(FITC)EPVHGV from HIV-1 reverse transcriptase. All peptides were used at a concentration of 10 *μ*M. rBA, relative-binding affinity; MW, molecular weight; HLA=human leucocyte antigen.

). Peptides that effectively bind to HLA-A^*^0201 are usually nonamers or decamers with the typical anchor position isoleucine (I) or leucine (L) at position 2 and valine (V), leucine (L) or methionine (M) as the C-terminal residue. The six highest scoring peptides, fulfilling these criteria, were synthesised and analysed for their binding affinity to HLA-A^*^0201 by a competition assay using peptide YLLPAIVHI from RNA helicase p72 as positive control and peptide ILK(FITC)EPVHGV from HIV-1 reverse transcriptase as reporter peptide. Binding affinities were classified as strong when the binding of a reporter peptide was inhibited by 75–100%, related to the inhibition of reporter peptide binding by a positive control peptide or as intermediate when the inhibition was 50–75%. The peptides 1472, 1479, 1487, 1494 and 2004 bound with high affinity, whereas peptide 1478 showed an intermediate affinity (Table 3). All six peptides were used for the *in vitro* stimulation of CD8+ T lymphocytes.

### *In vitro* generation of prostein peptide-specific and tumour-reactive CD8+ cytotoxic effector cells

CD8+ T lymphocytes isolated from the blood of two prostate cancer patients (donors 1 and 3) and one healthy donor (donor 2) were weekly stimulated with autologous DCs pulsed with a cocktail of the peptides 1478, 1479 and 2004. CD8+ T cells from the same healthy donor (donor 2) and an additional patient (donor 4) were subjected to stimulations with a cocktail of the peptides 1472, 1487 and 1494. After four stimulation cycles, T cells were tested for the presence of peptide-specific CTLs by chromium release assays.

**Figure 2:**
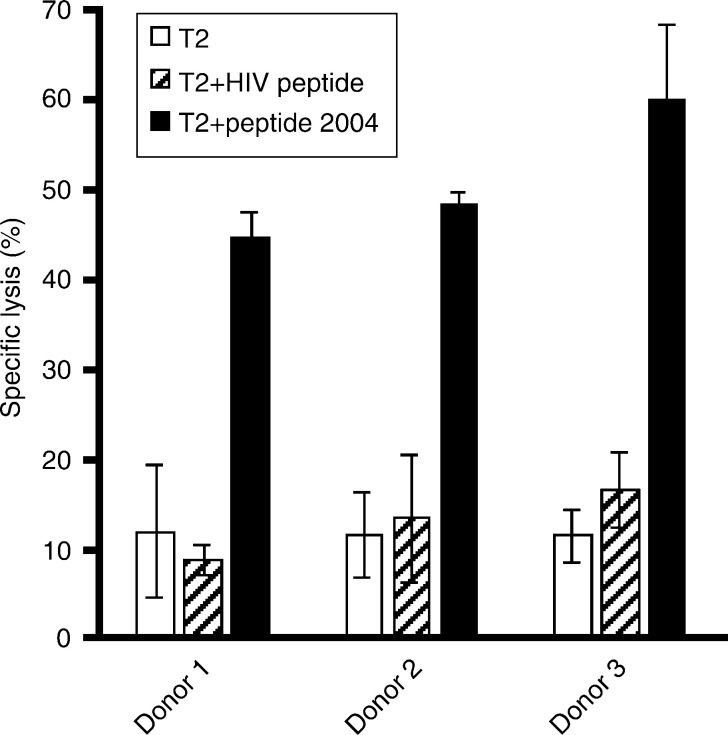
*In vitro* generation of cytotoxic effector T cells specifically recognising the prostein-derived peptide 2004. Purified CD8+ T lymphocytes of two prostate cancer patients (donors 1 and 3) and one healthy donor (donor 2) were weekly stimulated by peptide-pulsed autologous DCs. After four stimulations T-cell cultures were tested for the activation of peptide-specific tumour-reactive CTLs. The stimulated T-cell cultures were added to 3 × 10^3^ peptide-pulsed T2 target cells well^−1^ at an effector cell to target cell ratio of 20 : 1. Unloaded T2 cells and T2 cells pulsed with an irrelevant peptide from HIV reverse transcriptase served as controls. The results represent the mean values of triplicate determinations, bars indicate s.e.

**Figure 3:**
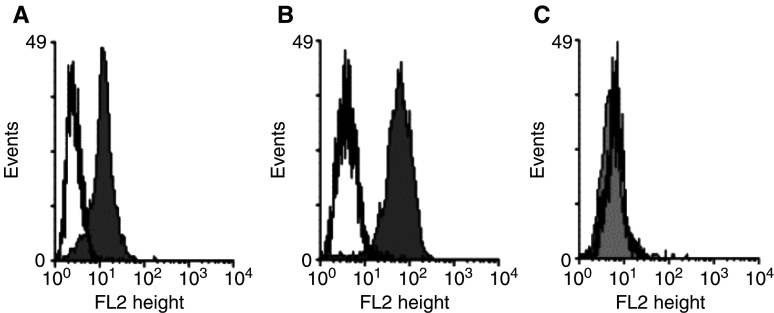
HLA-A2 expression on the surface of the tumour cell lines used as target cells in the chromium release assays. The PCa cell line LNCaP 1740 (**A**), the melanoma cell line 93.04A12.1 (**B**) and the HLA-A2-negative PCa cell line PC-3 as control (**C**) were analysed by flow cytometry using the anti-HLA-A2 antibody MA2.1 as primary antibody and a PE-labelled goat anti-mouse IgG F(ab′)_2_ fragment as secondary antibody. The solid line represents the IgG1 isotype control, the shaded peak represents the HLA-A2 staining.

**Figure 4:**
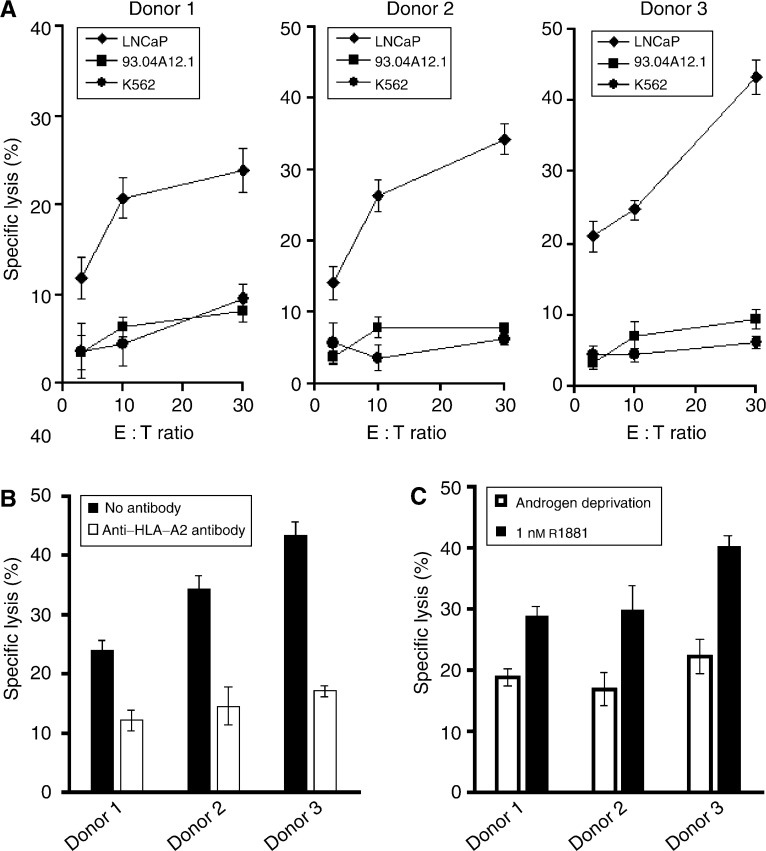
Prostein-specific lysis and HLA-A^*^0201-restricted recognition of LNCaP 1740 cells by the *in vitro*-generated cytotoxic effector cells. (**A**) After four rounds of stimulation activated CD8+ T cells from the three donors were cocultured with 3 × 10^3^ ^51^Cr-labeled LNCaP 1740, 93.04A12.1 or K562 tumour cells well^−1^ at various effector cell (E) to target cell (T) ratios (3 : 1, 10 : 1, 30 : 1). After 4 h of incubation, chromium release was determined. (**B**) The inhibition of T-cell-mediated cytotoxicity against LNCaP 1740 cells was tested in the presence of the monoclonal anti-HLA-A2 antibody MA2.1 at an E : T ratio of 30 : 1. (**C**) Influence of androgen deprivation or supplementation on the prostein-specific lysis of LNCaP 1740 target cells. LNCaP 1740 cells were cultured for 24 h in medium containing charcoal-stipped FCS. Cells were grown for additional 48 h in the androgen-deprived medium or in the presence of 1 nM R1881 and then used as target cells in a chromium release assay at an E : T ratio of 30 : 1. All results represent the mean values of triplicate determinations, bars indicate s.e.

Only peptide 2004 induced specific CTLs in all three donors tested (donors 1–3) as shown by the specific lysis of T2 cells loaded with this peptide (Figure 2

). Unloaded T2 cells and T2 cells pulsed with an irrelevant peptide from HIV reverse transcriptase were only marginally lysed (Figure 2). To determine whether peptide 2004 originates from intracellular processing of the prostein protein and is presented on the surface of tumour cells, the peptide-specific T cells were tested against the prostein-positive PCa cell line LNCaP 1740. This cell line expresses HLA-A2 molecules at a low density on the cell surface as demonstrated by flow cytometric analysis using the anti-HLA-A2 antibody MA2.1 (Figure 3A

). The melanoma cell line 93.04A12.1, which is negative for prostein transcripts (Figure 1A) but expresses HLA-A2 at a much higher level than LNCaP 1740 cells (Figure 3B), was used as a negative control. The T-cell cultures of all three donors effectively lysed LNCaP 1740 cells, whereas only marginal lysis of 93.04A12.1 cells was observed (Figure 4A

). Natural killer cell-like activity was excluded by the failure of the peptide 2004-activated T cells to lyse K562 cells (Figure 4A). As illustrated in Figure 4B, the recognition of LNCaP 1740 was restricted to HLA-A2 as shown by a significant reduction of lytic activity in the presence of the anti-HLA-A2 antibody MA2.1.

According to the observation that prostein expression in LNCaP 1740 cells is regulated by androgens (Figure 1B), we evaluated the cytotoxic activity of the *in vitro*-generated CD8+ effector T cells against LNCaP 1740 cells that were cultured in the absence or presence of androgen. As depicted in Figure 4C, the T-cell-mediated cytotoxicity of all three donors against target cells deprived of androgen was clearly reduced when compared with the lysis of LNCaP 1740 cells grown in the presence of R1881.

## DISCUSSION

Recently, prostein was identified as a novel protein with a unique specificity for normal and malignant prostate tissues as demonstrated by quantitative RT–PCR, Northern blot and cDNA microarray analyses at the transcript level and by immunohistochemical analysis at the protein level (Xu *et al*, 2001). Whereas Xu *et al* determined prostein expression in a collection of PCa tissues and normal prostate samples, we expanded this study by using matched pairs of tumourous and nontumourous prostate tissue for the mRNA quantification. Transcripts were abundant in both malignant and nonmalignant prostate tissues. Therefore, prostein like most of the so far described PCa-associated proteins is a tissue-restricted yet not tumour-specific molecule (Hubert *et al*, 1999; Nelson *et al*, 1999; Lin *et al*, 2000; Naz *et al*, 2002; Herness and Naz, 2003). When comparing the transript levels of prostein in the malignant tissues with the corresponding nontumourous samples, we found that prostein expression is not reduced in PCa tissues. This is an important prerequisite for molecules that are supposed to serve as a target structure for immunotherapy.

In recent years, HLA allele-specific peptides from a limited number of well-characterised prostate-associated antigens including PSA (Correale *et al*, 1998), prostate-specific membrane antigen (Tjoa *et al*, 1996; Horiguchi *et al*, 2002), prostate acid phosphatase (Peshwa *et al*, 1998; Inoue *et al*, 2001) and prostate stem cell antigen (Kiessling *et al*, 2002) have been identified as target structures of CTLs. These studies have clearly demonstrated that the immune tolerance against self-proteins expressed in normal prostate tissue can be overcome. In addition, clinical PCa responses have been described in vaccination studies based on CD8+ effector T cells targeting different TAAs (Murphy *et al*, 1999; Small *et al*, 2000). However, the heterogeneity of individual tumours (Klein *et al*, 2002) and the diversity of tumour escape mechanisms (Marincola *et al*, 2000) require the identification of additional target structures for T-cell-based immunotherapy.

The highly prostate-specific expression of prostein suggested extended evaluation of this protein for this form of immunotherapy. To identify CD8+ T-cell epitopes from prostein, six HLA-A^*^0201-binding peptides were predicted from the amino-acid sequence of prostein by a computer-based algorithm. For each of the peptides HLA-A^*^0201 binding was verified by a competition assay. Of this collection, only peptide 2004 (CLAAGITYV) encompassing the amino-acid residues 31–39 of prostein was able to induce specific CTLs *in vitro* when tested against peptide-loaded T2 cells. The lack of response to the other five peptides may indicate a low frequency or even absence of T cells displaying the respective specificities in the repertoire of the tested donors.

The CTLs specifically recognising peptide 2004 on T2 cells were also capable of lysing the HLA-A^*^0201-positive and prostein-expressing PCa cell line LNCaP 1740, demonstrating the autochthonous generation and presentation of this peptide by tumour cells. The prostein specificity and HLA-A2 restriction of LNCaP 1740 cell lysis was supported by different control experiments: (a) neither the natural killer cell-sensitive cell line K562 nor the HLA-A^*^0201-positive and prostein-negative melanoma cell line 93.04A12.1 was killed. (b) The lysis of LNCaP 1740 was markedly reduced in the presence of the anti-HLA-A2 antibody MA2.1.

The studies of Xu *et al* (2001) have clearly shown for both the transcript and the protein level that prostein is an androgen-regulated molecule. Here, we demonstrate that the killing of LNCaP 1740 cells by CTLs specifically recognising peptide 2004 is reduced when the target cells are grown in androgen-deprived medium and can be increased when the medium is supplemented with androgen. This effect can be explained by the androgen-regulated expression level of prostein that may directly influence the density of the respective HLA class I peptide complex on the surface of the LNCaP 1740 cells.

In summary, we identify an HLA-A^*^0201-restricted CD8+ T cell epitope derived from the prostate-specific protein prostein that was shown to be widely expressed in PCa. Our results emphasise this protein as a target molecule to be included in immunotherapeutic trials of PCa.
